# Associations between self-reported diabetes mellitus, disordered eating behaviours, weight/shape overvaluation, and health-related quality of life

**DOI:** 10.1186/s40337-019-0266-y

**Published:** 2019-11-01

**Authors:** Danilo Dias Santana, Deborah Mitchison, David Gonzalez-Chica, Stephen Touyz, Nigel Stocks, Jose Carlos Appolinario, Gloria Valeria da Veiga, Phillipa Hay

**Affiliations:** 1 Josué de Castro Institute of Nutrition, Federal University of Rio de Janeiro, Rio de Janeiro, Brazil; 2 Translational Health Research Institute, School of Medicine, Western Sydney University, Sydney, NSW Australia; 3 Centre for Emotional Health, Department of Psychology, Macquarie University, Sydney, NSW Australia; 4 Discipline of General Practice, Adelaide Medical School, University of Adelaide, Adelaide, South Australia Australia; 5 Adelaide Rural Clinical School, University of Adelaide, Adelaide, South Australia Australia; 6 School of Psychology, University of Sydney, Camperdown, NSW Australia; 7 Group of Obesity and Eating Disorders, Institute of Psychiatry, Federal University of Rio de Janeiro, Rio de Janeiro, Brazil; 8 Camden and Campbelltown Hospitals, SWSLHD, Campbelltown, NSW Australia

**Keywords:** Diabetes mellitus, Disordered eating behaviors, Weight/shape overvaluation, Health-related quality of life, Epidemiology

## Abstract

**Background:**

Eating disorders (ED) and disordered eating behaviours (DEB) have been found to be common in people with diabetes mellitus (DM). However, findings have been inconsistent.

**Objective:**

This study investigated the association between self-reported diabetes (Type 1 or 2) with ED/DEB (binge eating, subjective binge eating or loss of control overeating, severe dieting and purging) weight/shape overvaluation, and health-related quality of life (HRQoL) in a household survey in South Australia.

**Method:**

In 2017 2977 people aged ≥15 years, who were representative of the general population, were interviewed. Participants reported their gender, age, household income, highest educational attainment, area of residence, presence of DM, ED/DEB, level of overvaluation, current HRQoL and height and weight. For the analyses between ED/DEB, self-reported DM and HRQoL, a grouping variable was created: 1) people without ED/DEB or self-reported DM; 2) people without ED/DEB and with self-reported DM; 3) people with ED/DEB and without self-reported DM; and 4) people with ED/DEB and self-reported DM. Analyses were stratified by sex and age group.

**Results:**

Subjective binge eating prevalence was higher in people with self-reported DM (6.6% vs 2.8%, *p* = 0.016), and overvaluation was lower in those with DM (36% vs 43.8%, *p* = 0.007). In analyses stratified by sex and age group, subjective binge eating was higher in women and in people over 45 years with self-reported DM and overvaluation was lower in men and in people over 45 years with self-reported DM. However, these differences were not significant on tests of gender and age interaction. People in both DM groups scored significantly lower than people without DM groups on physical HRQoL. In contrast, people in both ED/DEB groups scored lower than people without ED/BEB on mental HRQoL.

**Conclusion:**

People with self-reported DM had a higher prevalence of subjective binge eating, a lower prevalence of overvaluation and there were no significant effects of age or gender. Furthermore, participants with self-reported DM and comorbid ED or DEB had impairments of both mental and physical HRQoL. Assessing an individual’s sense of control over eating along with other DEB is likely important for identification of these mental health problems.

## Plain English summary

Eating disorders (EDs) and disordered eating behaviours (DEB) such as binge eating and subjective binge eating (loss of control over eating small or normal amounts of food that are perceived as overeating episodes), severe dieting and purging, have been thought to be associated with diabetes mellitus (DM). Furthermore, recent studies have reported possible associations between DEB and factors related to DM, for example high body weight. In this study, we aimed to investigate the association between self-reported DM with ED/DEB, high body weight or shape concerns, and health-related quality of life in a household survey of older adolescent and adult Australians. We found that subjective binge eating prevalence was higher in people with DM, while a high body weight/shape concern was lower in these individuals of all ages and both men and women. In addition, individuals with self-reported DM and ED/DEB had poorer physical and mental and health-related quality of life respectively compared with people without these problems. We conclude it is relevant to assess an individual’s sense of control over eating, regardless of age or gender, along with other DEB for optimal mental and physical health care of people with DM.

## Introduction

Eating disorders (EDs) are disturbances of eating behaviors with a core psychopathology centered on eating, food and body image concerns [1]. There are four main types: anorexia nervosa (AN), bulimia nervosa (BN), binge eating disorder (BED), and also other specified or unspecified feeding or eating disorders (OS/UFED) according to the fifth edition of the Diagnostic and Statistical Manual of Mental Disorders (DSM-5) [1]. The estimated lifetime prevalence of eating disorders range from 1.1% (AN) to 4.4% (BED) in women [2] and less than 0.5% (AN) to 2.0% (BED) in men [3, 4]. The prevalence of OSFED and UFED has been less extensively investigated. However, it is estimated to be at least as high if not higher than AN, BN or BED [5, 6]. Furthermore, disordered eating behaviour (DEB) may occur in the absence of a formal diagnosis. DEB includes strict dieting or fasting, binge eating, or purging (for example, with laxative and diuretic misuse and/or self-induced vomiting) [7, 8]. Such behaviours are more common than full syndromes of EDs [9] and their frequency has increased considerably over the last years in different parts of the world [7–10].

EDs and DEB have been thought to be associated in various ways with diabetes mellitus (DM) [11–14], a group of metabolic diseases characterized by chronic hyperglycemia resulting from defects in insulin secretion, insulin action, or both. Most cases of DM fall into two broad categories. Type 1, characterized by an absolute deficiency of insulin secretion, or Type 2 conceptualized as a combination of resistance to insulin action and an inadequate compensatory insulin secretory response [15]. DM is an escalating health problem worldwide. The Australian Diabetes, Obesity and Lifestyle reported an increase in the prevalence of clinically diagnosed DM in Australians aged 25 years or older from 8.5% in 1999/2000, to 9.3% in 2004/2005 and to 12% in 2011/2012 [16–18]. A more recent survey investigating DM in Australia reported that 13.9% of non-Indigenous Australians had self-reported DM [19].

Recent studies have reported possible associations between DEB and metabolic, immunomodulatory and/or lifestyle factors related to Type 1 DM. Cherubini et al. [11] noted that the prevalence of DEB was 27% in boys and 42% (95% CI 31–53) in girls with Type 1 DM. A clinical profile of DEB was identified in these adolescents: overweight, little time spent in physical activity, low socioeconomic status, poor metabolic control, and skipping insulin injections. Furthermore, the probability of DEB increased 63% for every added unit of HbA_1c_, 36% for every added number of insulin injection skipped in a week and decreased about 20% for every added hour/week spent in physical activity. Other physical health comorbidities may also be important. For instance, Tokatly Latzer et al. [14] studying Type 1 DM and celiac disease in adolescents and young adults noted that the prevalence of DEB in the DM and celiac group was 3-fold higher than in the people with DM only or celiac disease only. This pattern was observed among both females and males. Conversely, Keane et al. [20] and Falcão and Francisco [21] did not find increased levels of DEB in young adults with Type 1 DM compared with a non-diabetic control sample.

DEB and EDs may also affect up to 40% of patients with Type 2 DM [12, 22], with BED being most common followed by BN [23]. Regular binge eating in individuals with Type 2 DM is common even in the absence of an ED diagnostic and is reported to be associated with higher rates of obesity. Notably, binge eating appears to be an independent risk factor for Type 2 DM, evidence indicating that in the vast majority of the cases, binge eating precedes the onset and is linked with significantly earlier age at the diagnosis of Type 2 DM [24]. Rates of DEB or EDs in people with DM can however vary widely. For example, a recent review has reported rates of BED between 1.2 and 8% in clinical samples of people with Type 2 DM [13]. Inconsistency in findings are likely because of different samples and methods used to determine DEB or EDs. In their review García-Mayor and García-Soidán also concluded that general population studies involving broad age groups are needed to clarify aspects of this relationship across the lifespan to avoid the selection bias of clinical samples [12].

Furthermore, since DEB have been associated with weight/shape overvaluation (i.e. excessive influence of shape or weight on self-evaluation) [25] it could be hypothesized that DM could also be associated with overvaluation. Some studies have investigated other body image constructs, such as body image dissatisfaction, in patients with DM. Falcão and Francisco [21] investigated young adults with Type 1 DM and their peers without DM and reported no significant differences between participants in relation to body image dissatisfaction. In contrast, Troncone et al. [26] studying children with Type 1 DM in a longitudinal study, found body size underestimation and dissatisfaction to be both prevalent and persistant over the 5-year study period. However, we identified no studies which have explored the associations between DM and weight/shape overvaluation.

We also aimed to explore associations between DM comorbid with DEB and mental and physical health-related quality of life (HRQoL). DEB, EDs and overvaluation are known to impact on mental HRQoL [6]. However, the additional impact from the presence of DM with DEB/ED is unknown. Thus, this article aimed to investigate the association between self-reported DM (including type 1 or 2) with DEB/EDs, weight/shape overvaluation, and HRQoL in a population-based sample of older adolescents and adults in a state of Australia. In addition, as DEB/ED may present distinct frequencies by sex [11, 14, 27] and age groups [24] in people with DM, we considered these may differ between men and women and young and old people, and thus we conducted a secondary exploratory study of these associations.

## Methods

### Sampling procedures

This is a cross-sectional study using data from the 2017 Health Omnibus Survey. This survey is conducted annually by Harrison Health Research under the auspices of the South Australian Health Commission. It comprises face-to-face interviews of a representative sample of the adult population in South Australia [28].

Metropolitan and rural ‘collector districts’ (530 out of 3939 in the state) were systematically selected based on a probability proportional to their size using as a reference data from the 2016 Australian census. Ten houses within each district were systematically chosen and the resident who had their birthday most recently and who was 15 years or older was interviewed. Up to six visits were made to each household (non-replacement samples). A pilot study was conducted to ensure participant understanding and feasibility of the questions. The participation rate in 2017 (completed interviews divided by the initial eligible sample minus non-contact after six attempts) was 65.3% (*n* = 2977).

### Ethics

Adult participants provided verbal rather than written informed consent, due to the practicalities of carrying out a large-scale survey and the low-risk nature of the survey content. For adolescents enrolled in the study (15–17 years old), written consent was obtained from the participant’s parent/guardian. The survey was approved by the University of Adelaide Human Research Ethics Committee.

### Measures

#### Exposure

##### Self-reported DM

Participants were shown a list of 20 conditions that included ‘diabetes/high blood sugar’ and if they ‘did not have’/‘did not know they had’ any of the investigated conditions the interviewer asked no further questions regarding these conditions. Those who indicated one or more conditions were then asked specific questions including: ‘*Has a doctor ever told you that you have diabetes/high blood sugar?*’ The answer options were ‘*Yes/No’.* Those who answered ‘no’ or ‘no condition’ on the preceding list were recorded as not having self-reported DM.

#### Outcomes

##### Disordered eating behaviors

Questions to ascertain the presence of these behaviors were based on the Eating Disorder Examination (EDE) [29], a structured interview used for ED diagnosis. Participants were asked whether they regularly (i.e. at least once per week over the past 3 months) engaged in (a) objective binge eating (i.e. eating an objectively large amount of food with a sense of loss of control), (b) subjective binge eating (i.e. eating was out of control when others might not agree the amount of food was unusually large, e.g. 2–3 pieces of bread), (c) extreme dieting (i.e. going on a very strict diet or fasting to control weight or shape), or (d) purging (i.e. use of laxatives, diuretics, or self-induced vomiting to control weight or shape). DSM-5 diagnostic categories were derived based on responses to these questions. The specific wording of the questions about these behaviours has been previously published [30].

##### Weight/shape overvaluation

This was measured through a question from the EDE [29]: ‘*On a scale of 0-6, where 0 is not at all important and 6 is extremely or the most important issue. How important an issue has your weight and/or your shape been to how you think about (judge or view) yourself as a person in the past 3 months? (It has been a really important issue to them, their self-esteem or their self-confidence*’). A score of 4 or more was used to indicate the presence of weight/shape overvaluation.

##### Health-related quality of life (HRQoL)

The Medical Outcomes Study Short Form questionnaire (SF-12) version 1 [31] was administered to all participants to measure HRQoL. Items assess impairment in physical and emotional health and the extent to which health status has limited quality of life in various domains (e.g., occupational, social, and other roles). Scores (ranging on a scale from 0 - 100) are transformed into two T-scores (physical and mental component summary scores, PCS and MCS), with a mean value of 50 and standard deviation of 10, with higher scores indicating a better HRQoL.

#### Covariates

##### Demographic information and body mass index

Demographic information collected included gender, age, household income, highest educational attainment and area of residence. Participants were also asked their height and weight, from which BMI (kg/m^2^) was calculated and were classified according to the World Health Organization [32] criteria: underweight = BMI < 18.5, adequate weight = 18.5 ≤ BMI < 25.0, overweight = 25.0 ≤ BMI < 30.0, and obese = BMI ≥ 30.0. For adolescents, BMI-for-age and sex z-scores were estimated and the following categories were used: low weight (Z-score < − 2); adequate weight (Z-score ≥ − 2 and ≤ 1); overweight (Z-score > 1 and ≤ 2) and obesity (Z-score > 2) [33].

### Data analysis

Survey data were weighted based on the correspondent sampling process and reweighted to the population distribution in 2016 [34]. All analyses were performed using SPSS (v.24). Descriptive statistics were generated for all demographic variables and chi-square (χ^2^) tests (for categorical variables) with Bonferroni-adjusted post-hoc z-tests were conducted.

Associations between each DEB or weight/shape overvaluation and self-reported DM were tested using binary logistic regression. Odds ratios (95% confidence interval) for such associations were calculated with reference category ‘not having DM’. All analyses were adjusted for BMI. Then, the same procedures were repeated with analyses stratified by sex and age group (15 to 44 and over 45 years) to evaluate differences in the relationship between DM and eating disorder features based on these variables. When different associations were identified in males or females or between young and old people, multiplicative terms between DM and sex or DM and age were included in the logistic regression models to test the heterogeneity of these associations (*p*-value for interaction).

For the purpose of this study, an ED/DEB variable was defined as participants who were identified with one or more of the ED features measured: objective and/or subjective binge eating, strict dieting/fasting, purging, with or without weight/shape overvaluation. Full syndrome disorders such as AN, BN, or BED were included in this analysis although they presented very low frequencies in the study population. Current (3-month) diagnoses were derived based on the questions regarding disordered eating behaviours and weight/shape overvaluation and were made according to DSM-5 criteria [1]. To facilitate analyses between ED/DEB, self-reported DM and HRQoL, a grouping variable was created with four categories: 1) no ED/DEB no DM (people without EDs and without DM); 2) no ED/DEB with DM (people without eating disorders and with DM); 3) ED/DEB no DM (people with eating disorders and without DM), and; 4) ED/DEB and DM (people with eating disorders and with DM).

Multivariate ANCOVA was employed with MCS and PCS scores as the dependent variables, ED/DEB and DM group as the independent variables, and BMI, gender, age, and educational attainment as the covariates. Where a significant main effect was observed, post-hoc pairwise Bonferroni-adjusted comparisons were used to compare differences between the categories of the ED/DEB and DM grouping variables.

## Results

The mean age of participants was 47.3 (SD = 19.0) years. There were slightly more women, with a household income greater than $60 k, and most were residents in a metropolitan region. The mean BMI was 27.0 (SD = 5.9) kg/m^2^ and 59.3% were classified as having overweight or obesity. The prevalence of DEB varied from 0.6% (purging) to 10.6% (objective binge eating), with 3.3% reporting subjective binge eating and 4.9% reporting strict dieting. Overvaluation prevalence was 42.9% and self-reported DM 11.3%.

Table 1 displays the sociodemographic features and BMI classification by self-reported DM. More people without DM were still at school (3.9%) compared with people with DM, none of whom were still at school. More people without DM were classified as adequate weight (41.4%) compared with people with DM (19.0%). In addition, people with self-reported DM presented a higher prevalence of obesity (44.9%) than those without DM (21.5%). A higher proportion of people with self-reported DM had a lower household income (60.9% vs 41.9%) and lived in the country (32.0% vs 24.5%) than people without DM.

**Table 1:** Sociodemographic features and BMI classification by self-reported diabetes (SRD) in the South Australian population, 2017

Variables	SRD	No SRD	*χ* ^*2*^ *(p)*	*Post-hoc*
	*n* (%)			
Gender			0.01 (0.905)	–
Male	166 (49.1)	1287 (48.8)		
Female	172 (50.9)	1352 (51.2)		
Household income^a^			**37.17 (<.001)**	–
Less than $60 K	176 (60.9)	849 (41.9)		**–**
More than $60 K	113 (39.1)	1078 (58.1)		–
Highest educational attainment			**15.08 (0.005)**	
Still at school	0 (0.0)	103 (3.9)		No SRD > SRD
Left school	126 (37.7)	887 (33.6)		**–**
Trade qualification	41 (12.1)	298 (11.3)		–
Certificate	91 (26.9)	678 (25.7)		–
Bachelor	80 (23.7)	670 (25.4)		–
Area of residence			**8.85 (0.003)**	
Metropolitan	230 (68.0)	1993 (75.5)		–
Country	108 (32.0)	646 (24.5)		–
BMI classification^b^			**103.49 (<.001)**	
Low weight	2 (0.6)	49 (1.8)		–
Adequate weight	61 (19.0)	1020 (41.4)		No SRD > SRD
Overweight	114 (35.5)	862 (35.0)		–
Obesity	144 (44.9)	530 (21.5)		SRD > No SRD

^a^Categorization of the variable was defined from the household income median

^b^BMI classification for adults: low weight (< 18.5 kg/m^2^); adequate weight (18.5–24.9 kg/m^2^); overweight (25–29.9 kg/m^2^) and obesity (> 30 kg/m^2^). For adolescents: low weight (Z-score < − 2); adequate weight (Z-score ≥ − 2 and ≤ 1); overweight (Z-score > 1 and ≤ 2) and obesity (Z-score > 2)

Table 2 displays the prevalence of DEB and weight/shape overvaluation by self-reported DM. Subjective binge eating prevalence was higher in people with self-reported DM (6.6%, *p* = 0.016), and overvaluation was lower in people with DM (36%) compared with people without DM (43.8%, *p* = 0.007). In analyses stratified by sex and age group, subjective binge eating was higher in women (7.1%) or in people over 45 years (6.0%) with DM than in women (2.5%, *p* = 0.027) or people aged 15–44 years (1.6%, *p* = 0.001) without DM. Furthermore, overvaluation was lower in men with DM (31.5%) than in men without DM (38.3%, *p* = 0.009). In addition, people over 45 years with DM presented lower overvaluation frequency (33.0) than people without DM (39.9%, *p* = 0.005) (Table 3). However, there was no evidence of a moderator role by sex or age, as the *p*-values for interaction were > 0.20 in all cases.

**Table 2:** Prevalence of disordered eating behaviors and weight/shape overvaluation in Australian people without and with self-reported diabetes, 2017

Variables	Self-reported diabetes		AOR (95%CI)^a^	*χ2 (p)*
	Yes	No		
Disordered eating behaviours	*n* (%)			
Objective binge eating	46 (13.6)	270 (10.2)	1.03 (0.71–1.49)	0.02 (0.883)
Subjective binge eating	22 (6.6)	74 (2.8)	**1.89 (1.13–3.18)**	**5.81 (0.016)**
Strict dieting/fasting	18 (5.3)	129 (4.9)	1.13 (0.64–1.98)	0.18 (0.675)
Purging	3 (0.9)	14 (0.5)	1.25 (0.37–4.26)	0.13 (0.719)
Weight/shape overvaluation	121 (36.0)	1152 (43.8)	**0.62 (0.48–0.80)**	**13.1 (<.001)**

^a^ AOR (95% CI) = Adjusted odds ratio (95% confidence interval): binary logistic regression (adjusted for BMI), reference category (no diabetes)

**Table 3:** Prevalence of disordered eating behaviors and weight/shape overvaluation in South Australian people without and with self-reported diabetes mellitus (DM) by gender and age

Variables	Self- reported DM		AOR (95%CI)*	*χ2 (p)*	Self-reported DM		AOR (95%CI)*	*χ2 (p)*	*p* ^*#*^
	Yes	No			Yes	No			
Disordered eating behaviours	Male				Female				
	*n* (%)				*n* (%)				
Objective binge eating	20 (12.0)	144 (11.2)	0.86 (0.51–1.45)	0.32 (0.570)	26 (15.2)	126 (9.3)	1.11 (0.65–1.89)	0.14 (0.707)	.218
Subjective binge eating	10 (6.1)	41 (3.2)	1.63 (0.77–3.45)	1.65 (0.198)	12 (7.1)	34 (2.5)	2.27 (1.10–4.69)	4.88 (0.027)	.508
Strict dieting/fasting	7 (4.2)	46 (3.6)	0.90 (0.39–2.09)	0.06 (0.803)	11 (6.4)	83 (6.1)	1.30 (0.61–2.79)	0.46 (0.498)	.664
Purging	–	3 (0.2)	–	–	3 (1.7)	11 (0.8)	0.44 (0.11–1.75)	1.34 (0.247)	n.a.
Weight/shape overvaluation	52 (31.5)	528 (41.2)	0.55 (0.38–0.79)	10.31 (0.001)	69 (40.1)	624 (46.2)	0.71 (0.49–1.02)	3.40 (0.065)	.623
	Age 15 to 44 years				Age ≥ 45 years				
	*n* (%)				*n* (%)				
Objective binge eating	12 (17.1)	168 (13.0)	1.11 (0.58–2.14)	0.11 (0.744)	34 (12.7)	102 (7.6)	1.24 (0.77–1.99)	0.80 (0.371)	.545
Subjective binge eating	6 (8.8)	53 (4.1)	1.72 (0.71–4.16)	1.46 (0.227)	16 (6.0)	21 (1.6)	3.28 (1.59–6.76)	10.35 (0.001)	.495
Strict dieting/fasting	6 (8.6)	88 (6.8)	0.78 (0.34–2.27)	0.08 (0.783)	12 (4.5)	41 (3.1)	1.49 (0.72–3.09)	1.15 (0.283)	.392
Purging	1 (1.4)	11 (0.8)	1.42 (0.16–12.45)	0.10 (0.750)	2 (0.7)	3 (0.2)	1.81 (0.29–11.46)	0.40 (0.526)	.718
Weight/shape overvaluation	33 (47.8)	619 (47.8)	0.86 (0.52–1.42)	0.35 (0.555)	88 (33.0)	533 (39.9)	0.64 (0.47–0.87)	7.87 (0.005)	.477

^#^Significance level for tests of interaction between male/female and young/old

*AOR (95% CI) = Adjusted odds ratio (95% confidence interval): Generalized linear models, binary logistic regression (adjusted by BMI), reference category (no diabetes)

Table 4 displays the associations between ED/DEB, self-reported DM and HRQoL, whilst controlling for BMI and demographic characteristics. A significant main effect of group was observed for both physical HRQoL scores (*F* (3, 2665) = 27.33, *p* < .001, partial η^2^ = 0.030) and mental HRQoL scores (*F* (3, 2665) = 30.94, *p* < .001, partial η^2^ = 0.034). Adjusted post-hoc pairwise comparisons revealed that both diabetic groups (i.e., no ED/DEB with DM, ED/DEB and DM) scored significantly lower than the non-diabetic groups (i.e., no ED/DEB no DM, ED/DEB no DM) on physical HRQoL (*p* ranged from .012 to < .001). In contrast, both eating disorder groups (ED/DEB no DM, ED/DEB and DM) scored lower than the non-eating disorder groups on mental HRQoL (*p* ranged from .001 to <.001).

**Table 4:** Adjusted mental (MCS) and physical (PCS) health-related quality of life scores in Australian people without and with diabetes and eating disorders or disordered eating behaviours (ED/DEB)

Variables	MCS	PCS
	Mean ± SE	
Without ED/DEB and without diabetes mellitus	52.84 ± 0.20^a^	49.23 ± 0.20^a^
Without ED/DEB and with diabetes mellitus	51.38 ± 0.58^a^	44.02 ± 0.57^b^
With ED/DEB and without diabetes mellitus	49.13 ± 0.49^b^	48.60 ± 0.48^a^
With ED/DEB and with diabetes mellitus	46.01 ± 1.28^b^	45.33 ± 1.26^b^

^a,b^Between-subject analyses were adjusted for the effect of body mass index, gender, age, and educational attainment. Main effects of group were observed for MCS and PCS scores. Differences in superscript letters indicate significant post-hoc pairwise comparisons on MCS and PCS scores, respectively (*p*’s ranged from .019 to < .001)

## Discussion

The first main finding of the present study was the association between self-reported DM and a higher frequency of subjective binge eating. This is consistent with other studies that have reported a higher prevalence of DEB in people with DM in an adolescent general population sample of people with Type 1 [11] and very high rates in a review of people with Type 2 DM [12]. A possible explanation for this association may be that a diabetic diet promotes guilt about eating what are ‘normal’ portions of food but are food types that are not approved for diabetic diets.

The second main finding was that people with DM had a  lower prevalence of overvaluation than people without DM . This has not been reported previously. However, some studies [21, 25] have investigated other body image constructs, such as body image dissatisfaction in patients with DM, and found conflicting results. Men and older people are at lower risk for EDs, particularly those characterised by overvaluation, i.e. anorexia nervosa and bulimia nervosa [6]. This may explain why men and older people had low weight and shape concerns but this did not reach significance on interaction analyses. It is also unclear why this should be the case in those with DM compared with people without DM. It does suggest that EDs which are not characterised by overvaluation, such as BED, may be a particular problem for people with DM. In addition, as DM in older individuals was associated with lower weight/shape overvaluation this is not likely to be a mediator for the higher presence of subjective binge eating In view of these findings, future research will be important in order to clarify putative associations between different aspects of body image disturbance (e.g., overvaluation, dissatisfaction, an/or preoccupation), DEB and DM.

With regards to the mental and physical HRQoL of people with ED/DEB and self-reported DM we found that both diabetic groups (i.e., DM with and without ED/DEB) scored significantly lower than the non-diabetic groups on physical HRQoL. In contrast both eating disorder groups (ED/DEB with and without DM) scored lower than the non-eating disorder groups on mental HRQoL. This is consistent with EDs and DEB being primarily a mental health problem while DM is primarily a physical health disorder. An individual with both problems thus has impaired mental and physical HRQoL. However, in this sample, there appeared to be no cumulative physical or mental health impact of having both disorders. This may be because DM was broadly defined and in this non-clinical community sample and the severity of both problems is likely lower than in clinical samples. This is also reflected in the small decrement of HRQoL overall.

The main limitations of our study are that we did not investigate diagnosed cases of DM, or compare Type 1 and 2 DM, or levels of DM illness severity. This limitation was due to the present study being part of a larger research, with already established datasets (i.e., variables were previously decided). Future research would benefit from prospectively developing research questions and methodologies to match these. Furthermore, using self-reported information may overestimate the number of cases. However, we examined DM broadly in order to increase the sensitivity of the data. Although we cannot exclude the potential bias of self-reporting, this is a commonly employed tool for DM surveillance programs [19, 35]. In this perspective, several surveys have reported good psychometric properties for DM self-reporting as an indicator of medically diagnosed DM [36, 37]. Other limitations included the small numbers of participants with purging, making analysis of this variable under-powered, and the use of self-reported weight and height to calculate BMI. However, high correlations of self-reported height and weight data with clinician-measured height and weight have been reported (e.g., Maukonen et al. [38]). Furthermore, it is important to comment that other lifestyle behaviours, such as low levels of physical activity, that may contribute to Type 2 DM and that increase with age were not investigated in this study. Future studies should examine these in the context of DEB to further clarify these relationships. Finally, as none of the findings of differences between males/females or young/old were supported by the statistical tests of interaction indicates they are likely the consequence of splitting the sample and caution should be applied to these results. Strengths of this study include the use of a large community-based sample, a selection of participants of both sexes, and a wide range of age groups. In addition, to date there are no studies that examined the relationship between DM and weight/shape overvaluation.

In view of the results found, some implications for public health and/or clinical practice arise. The findings suggest that associations between DM and DEB may differ across samples and may be stronger in clinical samples. Screening for ED and DEB in DM clinics and having referral pathways to ensure the ED is treated is important as associated poor mental health may impact on outcomes from DM. In addition, loss of control overeating as found in subjective binge eating was the only DEB found to be significantly associated with DM in this study. One implication is that clinicians should ask about loss of control over eating (no matter the amount consumed) as an important symptom and one that may be a useful screen for EDs in people with DM. The lower likelihood of overvaluation also suggests that in the general population an ED characterised by this symptom, e.g. anorexia nervosa, may be less common in people with DM than other EDs, e.g. BED. The lack of a significant effect for gender or age also suggests clinicians should consider ED/DEB in all people regardless of their age or gender. These community-based findings contrast with specialist DM and ED clinics where the young woman with Type 1 DM and anorexia nervosa with overvaluation driving ‘insulin skipping’ is a well recognised presentation [39]. However, future research is required to test these hypotheses.

## Conclusions

In conclusion, we found associations between self-reported DM with subjective binge eating and weight/shape overvaluation, where individuals with DM have a higher and lower prevalence of subjective binge and overvaluation, respectively. It is important to emphasize that no other study has reported DM and overvaluation associations and replication is required. In addition, we note that individuals with DM and comorbid eating disorder or DEB have impairments of both mental and physical HRQoL. Regardless of their age or gender, assessing an individual’s sense of control over eating, with other DEB, is likely important for identification of these mental health problems.

## Data Availability

The dataset used and analysed during the current study are available from the corresponding author on reasonable request.

## Abbreviations

AN: Anorexia Nervosa
BED: Binge eating disorders
BMI: Body mass index
BN: Bulimia Nervosa
DEB: Disordered eating behaviours
DM: Diabetes mellitus
DSM-5: Diagnostic and statistical manual of mental disorders
ED: Eating disorders
EDE: Eating disorder examination
HRQoL: Health related quality of life
MCS: Mental component summary scores
OS/UFED: Other specified or unspecified feeding or eating disorders
PCS: Physical component summary scores
SF-12: Study short form questionnaire
SRD: Self-Reported Diabetes
WHO: World Health Organization
